# Book Reviews

**Published:** 1831-09

**Authors:** 


### CRITICAL ANALYSES.

Quæ laudanda forent, et quæ culpanda, vicissim

Illa, prius, cretâ; mox hæc, carbone, *notamus.*—PERSIUS.

*Outlines of Physiology. With an Appendix, containing Heads of Lectures on Pathology and Therapeutics.* By WILLIAM PULTENEY ALISON, M.D. F.R.S.E. &c.—8vo. pp. 452. Blackwood, Edinburgh; Cadell, London.

WE remember reading in some old work, (which would have suited our friends of the Retrospective better than ourselves,) a most earnest wish that no one should be permitted to write a book who had not some new matter to communicate, and that none should be published as new on subjects already known. This monopolizing spirit of our blackletter exclusive would seem to haunt, and to have haunted, most authors from that day to this; for seldom do we open a volume without having our literary appetite palled by some excuse, which, even if good, we always think worse than none, and which seldom fails, whatever be its tenor, whether of barefaced pride or of ill-concealed assumption peeping from behind the skirts of affected humility, to leave an unpleasant impression upon the mind, or to dissipate the almost magic spell which a new book with a new name cannot but excite in all who, like us, are obliged to read and mark much that is neither creditable to write, nor profitable to peruse. Still, in sooth, we see not the occasion which so many apparently perceive of finding or feigning an excuse for doing that which is free to be done by all; and whether a student peruse the original book of nature, or abridgments thereof in the written book of man, or whether he consult short transcripts or the text in full, we know not wherefore the conclusions he arrives at, or the impressions he receives, should not, if it please him, be written in a book, and, if it please him, why that book should not be published: for, whatever may be said on either side, we feel convinced that authors write to please themselves, and to please themselves the public read. Tell us not of the student's midnight toil; we know it to be rather the midnight pleasure; and if the pangs of literary birth are ever felt in brains that labour, an author soon forgets the anguish, for joy that a book is borne into the world. It is true that in many works, as in many faces, much similitude is found; but even to this we do not think there can be made any rational objection. How seldom do men read a book a second time, and how little can they know of its contents from a first perusal? It is, therefore, to us a matter rather of congratulation than regret that the public are compelled to listen to a more than a three times thrice-told tale; so that, instead of lamenting the number of volumes on the same subject awaiting their perusal, they should rather be grateful for having the same story so often told, but in different words; and instead of considering the similarity of the facts as a matter to bewail, it should rather be valued as an evidence of their truth.

If amongst those who think great books great evils, we are not amongst those who think a multitude so many ills. What two persons ever saw the same prospect or the same statue from exactly the same spot, or received from the spectacle exactly the same impressions? Some direct their contemplation (the telescope of the mind) to one, some to another part; some view the statue in one, some in another aspect, it may be with glasses of different powers, or it may be the statue has a gold and a silver side; and thus the accounts, though somewhat similar, never precisely are the same, the similitude is sufficient to identify the objects, but the identity not sufficient to weary the beholder.

"Non omnibus facies una nec diversa," &c.

Hence we see not the occasion for apologies being preferred by authors for their works, on account of others on the same subject now existing: we would always thank them, and warmly, for shewing us a familiar prospect in a novel light, or even (if it must be so expressed) for cheating us into a reperusal of facts which we had often read before, remembering the truth of the pedagogue's quotation:

"Gutta cavat lapidem non vi sed sæpe cadendo,
Sic homo non doctus fit vi sed sæpe legendo."

But to the volume that heads our pages, and with which we are now more especially concerned: it opens with a very proper explanatory preface, although, upon the principles just developed, we differ decidedly from the author as to the belief expressed in the very first sentence it contains.

"Some apology may be thought necessary for the publication of a new elementary book upon Physiology, which contains no statements of new facts, within so short a time after the appearance of works of such value, on that subject, as the System of Dr. Bostock, the Outlines of Mr. Mayo, and the translations of Blumenbach's Physiology, by Dr. Elliotson, and of Magendie's Physiology, by Dr. Milligan." (*Preface*, p. i.)

Were any apologies necessary, those here offered would be certainly not ignored, viz. the attempt to reduce to system various insulated facts in physiology, and to furnish a text-book to the author's lectures: such reasons, however, we cannot esteem apologies, and no apologies are required to ensure a favorable reception to a volume so useful as the one before us.

The chief characteristic of these Outlines is "the attempt to give a more systematic form to the subject than has been usual in most recent publications;" and whether the reasonings are in every case correct, (which we much doubt,) or whether (as we suspect) they will require, in several points, to be hereafter modified, still the *facts*, which must always remain, will be more easily remembered by their association, even though the band should soon be obliged to be loosened, than were they merely presented to the reader in a form less methodized; for we fully agree with Dr. Alison, "that, in many of the medical writings of the present day, there is a want, not so much of facts in physiology, as of principles by which these facts ought to be connected, and by which the recollection and useful application of them may be best secured."

It is not to be expected that from a work which the author declares "contains no statements of new facts," we should be able to extract much that is novel for insertion in our pages. It will doubtless be found very useful for the purpose for which it was designed, viz. "as a textbook" to the author's lectures, and may also be read with advantage, although of course with less, by others, on account of its having been "thought right to enlarge, not on the subjects which occupy the largest portions of the lectures, but on those where, without such assistance from a textbook, the statements made in lectures may be most easily misapprehended; and, on this account, some of the subjects here discussed may be thought more abstruse than they might have been wished to appear."

An important feature, and that for which we expect this work will be chiefly valued, is the scholastic mode in which the propositions are supported, if not always proved: but this scheme of marshalling evidence unavoidably entails a mannerism which at length, especially for the general reader, becomes tedious, and deprives the subject of much of the beauty and enchantment which a style less rigid would admit; so that, if acknowledged as a work of equal importance, it will be read as one of far less interest than the Outlines of Mr. Mayo.

It has been too long the fashion to misconstrue the word, and to misstate or misconceive the object of, metaphysics. Milton once feigned metaphysical researches to form part of the tortures of fallen spirits; and persons in general seem to have shunned such abstract studies, as if they feared to touch an unholy thing. This prejudice, however, is waning fast, and writers on physiology, among others, more and more extend their researches into the principles of knowledge and the powers of the mind. This is a topic replete with interest, and is discussed by Dr. Alison in his thirteenth section, "of the Mental Faculties," though less fully than we could have wished, yet with very considerable ability.

After some preliminary observations on this subject, he there remarks:

"The exercise of our senses naturally and immediately leads us to form certain general ideas or notions, the tendency to the formation of which has been called by some metaphysicians, the Power or Faculty of Simple Apprehension; and, in connexion with these, we have a natural disposition to put faith in certain propositions or principles, which have been called by Dr. Reid, Principles of Common Sense, and by Mr. Stewart, perhaps more correctly, Fundamental Laws of Human Belief; of which no proof can be given; which we know not from Reasoning, nor from Experience, but by Intuition; and our confidence in which must be stated as an ultimate fact in the constitution of our minds. A little attention to the existence, and to the authority, of these laws of belief, will enable us to set aside a great deal of useless controversy, and simplify our account of the mental part of our constitution.

"Acquiescence in these principles is, in fact, equally necessary in the prosecution of physical, or even of mathematical, science, as of metaphysics; but in the former sciences, they have been always tacitly admitted; and only brought into question in the latter, because it is only in the latter that the instruments by which truth is sought are themselves made subjects of discussion.

"But as we have no instruments to apply to inquiries into the mind itself, different from those which we employ in the other sciences, we must be content to use them in the same manner, and trust them equally; otherwise we must abandon the labour." (P. 276.)

And shortly afterwards continues:

"It is only by careful reflection on the circumstances in which these articles of belief take possession of our minds, that we can judge on this point; but the following have been stated as the most distinctive marks of those principles, which are entitled to be regarded as fundamental laws of human thought.

"1. These principles are formed, and in the first instance believed, uniformly, by all men, and in all circumstances, and do not appear to be influenced by any accidents of situation, habits, or previous associations of individuals.

"2. They are of such a nature, that they can neither be attacked nor defended, in any other way than by reference to principles, which are not more universal, and have no other foundation than themselves.

"3. Their practical influence is found to extend, even to those who affect to question their authority.*

"Judging by these marks, and by carefully attending to the natural, uniform, and irresistible tendency of our own minds, we believe that the following principles, so formed, may be regarded as belonging to the class of fundamental laws of belief.

"1. The belief in the existence of the sensations and thoughts which pass through our minds, and in our own existence, as sentient and thinking beings, or the faith we repose in the Evidence of Consciousness.

"2. The belief which we repose in what we remember to have felt or thought, or in the Evidence of Memory, and in our own Identity during the whole time to which our memory extends, which implies the formation of the general idea of Time.

"3. The belief, already mentioned, in the external and independent existence of the causes of our sensations, or in the Evidence of Sense, which involves the formation of the general idea of Space.

"4. The belief in the existence of an Efficient Cause, for the changes that we see take place around us, which implies the formation of the general idea of Power.

"Here it is to be observed, that our senses never inform us of any events in nature as being causes of other events, in any other way than by shewing that they are immediate and invariable antecedents of these. It is only by experience of the consequences which follow any change, that we learn what consequences to anticipate, when we see the same change again.

"But although this be the case, most philosophers have thought that the mere observation of changes in the world around us naturally suggests to the mind, not only the facts of antecedence and consequence, but farther, a general notion or idea, which we express by the term Power; and the notion thus expressed, being truly an attribute of Mind, is considered as the first step in Natural Theology.

* See Stewart's Elements of the Philosophy of the Human Mind, vol. ii. ch. i. art. 2 and 3.

"5. The belief in the stability of the Order of Nature, which is necessary to enable us to apply the experience of the past to the conduct of our lives, or the knowledge of natural phenomena acquired by one person, to the instruction of others. This belief is judged not to be the result of experience, because it may be observed to operate the most strongly in those whose experience is most scanty, and in relation to subjects which are perfectly new to them.* Indeed, several philosophers teach that our belief in the independent existence of external things, being founded on a repetition of sensations, is resolvable into this principle of our nature.

"6. Our belief in our own Free Will, involving the general idea of Voluntary Power, in like manner attends a part of those mental changes, which the exercise of sensation excites.

"In regard to this, just as in regard to the other principles now stated, it is a fair question, whether there is any reason for thinking that the feeling of voluntary power, which undoubtedly attends certain acts of the mind, can be fallacious. But if no irresistible reason be stated against the belief, it is unreasonable and unphilosophical for us to ask, because it is impossible for us to obtain any other reason for the belief, than the fact, that in performing certain of the mental acts, of which we are conscious, we are naturally and uniformly impressed with the conviction that we may do so or not as we please. If no clear proof be given that this conviction is erroneous, and that the general notion of free will which it involves is a fallacy, all that we have to do is to state, in the course of our account of the different mental phenomena, what the circumstances are in which it uniformly and spontaneously arises in our minds.

"What has already been said of Sensation, and of these different acts of Thought which naturally and inevitably succeed sensation in every individual of sound mind, is sufficient to shew that the only foundation of much of our belief, and the only source of much of our knowledge is to be found in the constitution of our own minds. Not only are the notions that we form of the qualities of external things, which affect our senses, totally different from our sensations themselves, but certain general ideas, which inevitably arise in our minds, in consequence of the exercise of our senses, are at once perceived, when the attention is fairly fixed on them, to have an extent of application far beyond what the senses themselves can ever reach. The simple notion of our own existence, which is sufficiently suggested to our minds by the occurrence of any single sensation, instantly extends to the whole period of which our memory gives us any information. The notion of Power, or Causation, which may be suggested by the observation of a single striking change in the world around us, is immediately judged to be applicable to all changes without exception. The notion of

* See Stewart's Elements, vol. ii. chap. i. sect. 3.

Time is no sooner formed, than it swells in the human mind to Eternity, as surely as the notions of Space and of Number to Infinity.

"Even from these simple instances, it appears how necessary an addition was made to the scholastic maxim "Nihil in intellectu quod non fuerit prius in sensu," by the words "Nisi intellectus ipse;"* and how extended a meaning we must annex to the word Reflection, if we assent to the doctrine of Locke, that all our knowledge is derived from Sensation and Reflection. Although 'sensible objects may be the destined medium to awaken the dormant energies of the understanding, yet are these energies themselves no more contained in sense, than the explosion of a cannon in the spark that gave it fire.'" (P. 278.)

We cannot pretend to follow the author through the subsequent portions of this essay, and only offer the above as samples of his style and mode of reasoning, which are close and in general conclusive. The establishment of first principles is, however, the most important and difficult step in such researches: there was little exception to be taken to Des Cartes after he confessed himself convinced of his own existence, of which, however, if he had ever truly doubted, his enthymeme, "cogito ergo sum," was little calculated to persuade him; for self-evident propositions, as they the least require, so are they, of all authorities, the least susceptible of proof.

But we have done; and we restrain ourselves abruptly, well knowing that it is easier not to enter upon so entrancing a topic, than to leave off within reasonable limits when hot in the pursuit of an idea which, with an old episcopalian, we could love to hunt through the world till, to escape our grasp, it should be compelled to leap into infinity.

---

### *Auscultation the only unequivocal Evidence of Pregnancy: with Cases.* By John C. Ferguson, A.M. M.B., Fellow of the King and Queen's College of Physicians in Ireland, &c.†

This is an extremely interesting paper upon a subject which has only recently attracted the attention of the profession, and which well deserves further and diligent investigation. Dr. Ferguson remarks, that, amidst the varied and important applications of mediate auscultation, which have been so zealously prosecuted, there still remains one which has not yet received a due degree of attention, nor been sufficiently

* Leibnitz.

† Dublin Med. Transactions, vol. i. part i. New Series.

submitted to the test of experience; its employment in the detection of pregnancy, and the incontrovertible evidence it affords of a living fœtus in utero.

It must be admitted, that all the signs of pregnancy that have been observed and enumerated by systematic authors, and are looked for by practical accoucheurs in the formation of a diagnosis, are fallible, equivocal, and deceptive; capable of being simulated not only by a state of the uterus, very different from pregnancy, but by many abdominal diseases. Dr. Gooch, for instance, in his chapter on the symptoms of pregnancy, states, "it is clear that if these symptoms always accompanied pregnancy, we should always know when it existed, and if they never accompanied any other state, we could never mistake any other state for pregnancy: but unfortunately they possess neither of these requisites for infallibility: they may be absent in patients who are pregnant, and present in those who are not so, and thus give rise to frequent errors." Dr. Ferguson admits that the subject upon which he writes still requires much maturation; that much is still due to society, and our profession, by those who have extensive opportunities of observation. For the last three years he has examined by the stethoscope every pregnant woman who presented herself, under any circumstances, at either of the public institutions to which he is attached: he has thus had opportunities of testing the value of mediate auscultation in such cases above one hundred times, and *in every instance, with but one exception,* he could detect either pulsation of the fœtal heart or placentary noise, generally both, after the patient had passed the fifth month of gestation, and in many, indeed the majority, before that period. In the individual case excepted, he could detect neither the one nor the other; but he had no opportunity of making a second examination, "which should never be omitted in doubtful cases," for the woman, contrary to her promise, did not return to him.

In not more than eight or ten cases was it found necessary to remove the usual clothes of the patient from beneath the instrument, leaving only the chemise between it and the abdominal parietes; nor to oblige them to assume the horizontal position, "which is decidedly more favorable to an accurate examination than the erect or sitting." Dr. F. usually examines the woman while seated in a chair, and without the removal of any part of her dress.

The two signs of pregnancy which the stethoscope affords were first observed by M. Kergaradec.

"He pointed them out to the immortal author of Mediate

Auscultation; and often have I seen Laennec practically confirm their truth, and heard him acknowledge their value: they consist in what has been termed the 'bruit placentaire,' and the pulsations of the fœtal heart. I believe they are not in general observed, for obvious reasons, before the fifth month; but after that period an accurate observer will seldom fail to detect either, and in most cases, both. The placentary noise, which is nothing more, as I conceive, than 'bruit de soufflet' in the arteries connecting the placenta with the uterus, should be sought for in either Iliac region, where, at least according to my experience, it will be most generally found. Yet have I detected it in almost every point of the abdomen, nor does it ever vary from the place where first heard.

"The only error that I am aware of, into which we are liable to fall in making this examination, is, where the pulsations of the iliac arteries are accompanied by a 'bruit de soufflet.' This of course is well calculated to deceive; but when the artery is the source of the bruit, we shall hear it, I am inclined to think, equally on both sides, at least I never met a case where this did not hold. Besides it will not be heard, save in the groin; whereas the noise of the placenta is heard over a space of some extent, perhaps three or four inches square." (P. 69.)*

Dr. F. has heard the fœtal heart in almost every region of the abdomen: although it and the placenta are sometimes heard on the same side, and he has even found them on the same spot, yet he has observed that they are to be detected, in the majority of cases, at opposite sides, generally in the iliac region; so that this is the situation he always examines first, and there is rarely occasion to shift the instrument to any extent before either one sign or the other is discovered. The fœtal heart, however, unlike the placenta, is not always heard in the same place in the same individual. Its double beat is well marked, and the frequency of its pulsations are, Dr. F. believes, always much greater, often double, that of the mother's heart.† As a proof how small a fœtal heart may be distinctly heard by the stethoscope, the following fact is related: The heart did not exceed the size of a hazel nut, and yet the two sounds were detected under these circumstances.

"A goat had been procured for a very different purpose by Doctors Hunt, Corrigan, and myself, and bound on its back on the

* In our last volume, p. 258, we gave an analysis of an interesting paper on the Placental Soufflet, by Dr. E. Kennedy.—Editor.

† "Since writing these remarks, a case occurred to me, in which the fœtal heart was distinctly heard to beat only twenty-eight, the mother's one hundred. I also observed an irregularity in its rhythm. Dr. Corrigan, who was with me when I examined this patient, satisfactorily verified this observation. I am not aware that any similar fact has been as yet recorded."

operating table. I casually applied the stethoscope to its abdomen, without the slightest previous knowledge of its pregnancy, and was surprised to detect, almost immediately, the distinct double pulsations of a fœtal heart. My two friends, to whose accuracy of observation I have been often indebted, satisfied themselves perfectly of the fact, and, on examining the uterus about an hour afterwards, we extracted a fœtus, of which the minute preparation which I now offer was the heart. On inquiring from the person who sold us the goat, on whose accuracy we could depend, we learned that it was exactly seven weeks from copulation." (P. 70.)

To illustrate the practical value of the physical signs of pregnancy which he has enumerated, Dr. F. relates six cases, of which we shall now proceed to give a brief account.

Case I. The patient was anxious to conceal her pregnancy, and for some time deceived the author by a well-sustained story of dyspeptic ailment. She at length equivocated in her account, and suspicions were excited: the stethoscope was applied, and "the riddle was soon read." The heart of a fœtus was almost immediately detected, and also the placenta; "nor, which is a matter of some moment in such cases, had the patient the slightest suspicion of the object of such an investigation." The young lady received the announcement of her being pregnant with great indignation, and with well-feigned agony at the very idea of her virgin innocence being suspected; and, says Dr. F., "had I not the positive evidence of my senses to confirm me in the opinion I had expressed, I should have felt extremely uncomfortable at the situation in which I found myself." She at length confessed she was in her fifth month.

Case II. The patient, eighteen years of age, stated that her general health had been declining for twelve months, but only during the last two months had she observed the swelling of her belly; and this her married sister assured Dr. F. at times almost entirely disappeared. On finding the enlargement of the abdomen over the pubes to be of a firm, unyielding nature, immediate recourse was had to the stethoscope, without any notice being given of the object in view. The fœtal heart was heard to beat 136 in the minute on the first application of the ear to the instrument: the mother's pulse was eighty. Dr. F. unceremoniously stated his opinion to the patient in private: she denied stoutly, wept bitterly,—and was safely delivered three months after he first saw her.

Case III. is too interesting to abridge.

"In December 1828, business brought me sixty miles from Dublin, where I met in consultation an eminent and intelligent practitioner. He related to me some cases of interest then under his care; but one in particular, he expressed a strong wish that I should see with him. His patient was the daughter of a respectable shopkeeper; her apparent disease ascites; but from what organic cause was the point most difficult to be ascertained. The girl's history of herself, stating that her ailments had been of twelve months standing, that her abdomen had been occasionally enlarged during all that time, though latterly more permanently so, that the remedies which had been prescribed for her had produced the most beneficial effects, was all confirmed by the testimony of an anxious and intelligent mother. To enter more fully into the particulars of this well got-up, and admirably acted drama, would be more tiresome than interesting; suffice it to say, that the agitated and confused manner of this young woman, when I was first introduced to her, struck me forcibly. This, added to the peculiar shape and solidity of the belly, immediately roused a suspicion, which at the moment, I confess, I thought it unjust to entertain. However, I knew well the advantages I possessed in mediate auscultation, and resolved to avail myself of them. Unfortunately I had not a stethoscope with me, nor could one be conveniently procured; thus circumstanced, I bethought myself of Laennec's original idea, the roll of paper, which I used to some effect. With considerable difficulty and embarrassment, I succeeded in discovering the pulsations of the fœtal heart. From the nature of the instrument, they were of course feeble and indistinct, yet sufficiently evident to leave not a shadow of doubt on my mind as to the organic cause of the ascites.

"I regretted not having my stethoscope, more particularly as it prevented me from pointing out to my friend those peculiar phenomena which I have no doubt, with the instrument, he could have detected, and which must, of necessity, have been followed by conviction in his mind. The announcement of my diagnosis surprised him: he was, and under all the circumstances, I admit with apparent reason, rather incredulous. As a proof of his still leaning to his own opinion, and doubting mine, in letters which passed between us on other matters, he often expressed a hope that his patient was improving; but kindly added, that he was not inclined to think the less of the value of auscultation, on account of the peculiarity of the circumstances under which I had formed my diagnosis. I felt satisfied, however, that the day was not far distant when he would change his mind, and I extract the following from a letter which I received in reply to one I lately addressed to him, making some inquiries into the particulars of this case.

"'An accouchment has fully confirmed your diagnosis, which I confess has contributed to raise in my esteem, not merely the discriminating qualities of the stethoscope, but also your tact in using it, which was on that occasion brought to a very nice trial, as, in want of an instrument, you were obliged to make use of a few sheets of coarse wrapping paper as a substitute; so that even had the positive assertion of pregnancy which you then made to me, turned out in the event to be erroneous, I should have imagined that the rudeness of the instrument employed in your examination would have furnished you with more than sufficient apology for the error. As it turned out, however, this imperfection of the instrument has only contributed to increase my desire to become acquainted with its use and application.'

"Thus this eminent general practitioner, of long and extensive experience as an accoucheur, was completely led astray by the well-feigned symptoms of this girl, and I believe never for a moment even suspected her pregnancy; nor do I hesitate to express my conviction, that no man who saw her, heard her history and knew her respectability, would have been justified, relying solely on the usual symptoms, in hazarding such an opinion.

"I may be told that such mistakes are not common; that they not only do not, but could not occur to men of acuteness and matured judgment. Both these assertions seem to me to be without foundation, and to merit a distinct contradiction; the former, because so many cases of this nature having in so very short a space of time occurred to myself, whose opportunities of meeting them have been so limited, I must naturally infer that to others they are by no means of rare occurrence; the latter, because I have myself seen in this city, not to leave home, two cases which were believed to be ascites, treated as such by clinical professors, up to the very day of their accouchment. Nor did either of these two gentlemen suspect the truth, or if they did, could not, of course, have possessed the means of putting the matter beyond doubt; yet the acuteness and judgment of both, few who know them will question. Besides, how many similar cases have we not all heard of, and on the most undoubted authority? Have we not even cases recorded, where the want of a sufficient guide, under similar circumstances, has led men of eminence so far astray, that the very fœtus in the womb has been, I believe in more instances than one, perforated by the trocar? But let us look to a far more awful consequence of uncertainty in such cases, and yet one of infinitely more frequent occurrence. Do we not daily read accounts of unfortunate infants being either exposed or inhumanly murdered? And is it not an acknowledged fact, that those which become the subject of public investigation are but a small proportion of the number of infanticides which really take place? Is it not also undeniable, that it is by females such as these I have described, that attempts are made on the lives of their children, either in utero or after birth? And if it be so, what a check must be given to this murderous, but too common system, by medical men possessing the means of pronouncing positively on a case of pregnancy; and thus, by being able to apprise friends of the real fact, do away with all further motives for secrecy on the part of the patient; for to the hope of avoiding detection is to be attributed, I believe, all the melancholy sequelæ of concealed pregnancy." (P. 74.)

It is not the mere detail of the circumstances of this case which alone claims our attention: the remarks which Dr. Ferguson appends to it are equally important and true, both in relation to the frequency of mistaken opinions in such instances, and to the melancholy consequences which have so often resulted from our want of means to determine positively the existence of pregnancy.

Case IV. This patient had been married three years, and had had no children; the menses had ceased for five months; she suffered from headach, want of appetite, and debility. Several medical men had treated her for the removal of these symptoms: neither they nor the patient herself had ever suspected the existence of pregnancy; she, indeed, "positively protested against its possibility," for what reason could not be imagined. On examination, a fœtal heart was heard to beat 130 distinctly, the mother's, ninety; the placentary noise was also evident. The accuracy of the diagnosis was proved by the accouchment of the patient at the proper period.

Case V. affords a striking illustration of what Dr. F. would particularly urge on the attention of the profession, namely, "that the absence of the pulsation of the fœtal heart and placentary noise, amounts, if not to positive, at least to presumptive proof of the absence of a living fœtus."

Case VI. The first application of the stethoscope to the left iliac region detected a fœtal heart beating 120; the mother's, ninety. It was probable that the patient was in her seventh month of gestation. To all who would say that a pregnancy so far advanced could be ascertained without the aid of mediate auscultation, Dr. F. would put the two following queries:

"After reflecting on the many errors which have been committed through a dependence on the common signs of pregnancy, could you take upon yourself the awful responsibility of blasting, with her friends and the public, the fair fame of any female? Or could you, if appealed to, confirm such an opinion by a conscientious oath? I am persuaded a negative reply must be given to both questions." (P. 87.)

In this instance, again, the vast importance of ascertaining to a certainty the existence of pregnancy, is shewn. The patient had endangered her own life, and attempted that of her unborn offspring, and in all probability, as Dr. Ferguson remarks, had her pregnancy remained concealed from her friends until after the period of her accouchment, she would have endeavoured to cloak her fault by the perpetration of the most heinous and unnatural crime.

"Thus, then, I conceive it to be sufficiently established, that either a placenta or fœtal heart being distinctly heard constitutes infallible evidence of pregnancy; evidence on which a medical man may, if required, conscientiously and positively swear to the fact; which I believe all admit, and our legal records shew, could not be done under ordinary circumstances. But the more difficult question, 'Are we, in the absence of both signs, to infer the non-existence of pregnancy?' still requires more consideration and further investigation. At present I shall merely state my own opinion, formed, I admit, on a limited observation of facts; it is this, that where the patient has passed the fifth month, and in the great majority of instances before this period, on an examination of the abdomen carefully conducted by a person well practised in stethoscopy, and, if necessary, repeated twice or thrice at intervals of a day or two, a fœtal heart or placentary bruit, or both, will, in every instance, be detected. I am well aware that many will conscientiously dissent from this opinion: but I would beg leave respectfully to remind those who do, of what I am satisfied they themselves must have experienced, that to detect these signs with facility, requires long practice, considerable tact, and a most acute stethoscopic ear, requisites, perhaps, not yet possessed by all who employ, and some who, too often without good cause, condemn the instrument. However, let us suppose a case of doubtful or suspected pregnancy, and that a practitioner being called upon to give a diagnosis, can detect neither sign, I may be asked, 'How is he to act?' He is unquestionably in no greater difficulty, than before he sought for them, and he may then have recourse to other means to satisfy himself, and guide him in forming his opinion; he may refer to every general symptom of pregnancy ever mentioned by author or practitioner, and giving them all, both individually and collectively, their due value, shall his doubts, I would ask, be then changed to certainty; shall he, relying on them alone, be prepared to make oath on the presence or absence of a fœtus? Assuredly not. But to reply to the question put to me, 'How is he to act?' I conceive that, so situated, his diagnosis should be peculiarly cautious and circumspect; and though symptoms and circumstances be strong enough to induce others to lean to the opinion of pregnancy, if he have a well founded confidence in his tact as an auscultator, in my mind he should in no instance hazard the adoption and expression of such an opinion; but on the contrary, he should state honestly his doubts, repeat his examinations at intervals, and in every possible manner, and if unsuccessful in his search, considering the absence of these signs as strong evidence of the absence of a fœtus, lean to the side of mercy, and await the issue.

"I would then, in conclusion, respectfully submit to the profession, that an audible fœtal heart or placentary bruit, may be considered as 'infallible' evidence of a fœtus in utero; and also, that the absence of these phenomena amounts, if not to positive, at least to presumptive proof of the contrary." (P. 80.)

Dr. Ferguson concludes his paper by calling the immediate attention of that branch of our profession whose more peculiar province it is, to this interesting and important subject. This appeal "is made in the cause of science, in the cause of humanity, and by one who has no object in view save the advancement of truth, and the best interests of his profession."

No one can doubt the purity of Dr. Ferguson's motives in giving this communication to the profession; nor can it be denied that the evidence he adduces is very satisfactory,—we had almost said, demonstrative of the facts for which he contends. Many practitioners are still inclined to ridicule the use of the stethoscope, or at least to deny that its application can add any thing to our diagnostic knowledge of disease, which may not be obtained by a careful examination of symptoms; but if accumulated experience should prove, as we firmly believe it will, that by its assistance we can, at the fifth month of utero-gestation, or, perhaps, before, determine positively, which we *cannot do* by any other means, whether a female is or is not pregnant, and, *still more*, whether the fœtus in utero is living or dead, it must then be regarded as one of the most useful instruments ever invented, even if its employment should be entirely abandoned in all other cases. We shall certainly omit no opportunity of satisfying ourselves upon these points by repeated experiments; and we feel confident that no practitioner of midwifery, who has the interests of his profession at heart, will fail to assist the highly important investigation to which Dr. Ferguson has so powerfully contributed by his valuable essay, which does him great honour, not only from the practical instruction it conveys, but from the philanthropic spirit which has evidently animated his inquiries.

*History of the Epidemic Spasmodic Cholera of Russia; including a copious Account of the Disease which has prevailed in India, and which has travelled under that Name from Asia into Europe. Illustrated by numerous Official and other Documents, explanatory of the Nature, Treatment, and Prevention of the Malady.* By Bisset Hawkins, m.d., Fellow of the Royal College of Physicians, &c.—8vo. pp. 306. Murray, London.

*Cholera Morbus. A short and faithful Account of the History, Progress, Causes, Symptoms, and Treatment, of the Indian and Russian Cholera, taken from various authentic Sources; with Cases as related by Practitioners in India; and also the interesting Case lately given to the Public by* Dr. H. Roe, *of the Westminster Hospital.* By John Austin, Surgeon.—8vo. pp. 44. Hughes, London.

Although no facts have as yet transpired which can, if coolly considered, lead to the conclusion that cholera has been recently either more frequent or more fatal in this country than it has often been before at this season of the year, and although it is the opinion of those who are the best judges of the subject, that it is very improbable that the disease will ever prove formidable in an English climate, still it is incumbent upon all medical practitioners to be prepared for the worst, by taking advantage of the unfortunately too great experience of this dreadful malady which has been acquired in other countries.

The work of Dr. Hawkins contains the substance of all that has lately been published upon the subject, in a concise and convenient form for reference: he first gives an account of the regulations issued, and of the measures adopted, by the Russian government, to check the progress of cholera, and to protect individuals.

The second chapter consists of an extract from Mr. Annesley's excellent work on the Diseases of India, relating to the preservative regimen that was recommended in India against an attack of epidemic cholera.

"Under this head may be comprehended the general injunction of avoiding the predisposing and exciting causes of the disease. Whatever tends, directly or indirectly, to debilitate or fatigue the system; whatever lowers its vital energy, as excesses of every description, disposes to the operation of the efficient cause of the malady. On the other hand, I am fully persuaded that whatever tends to preserve this energy, serves to render the system impregnable to its operations.

"Exposure to cold, to chills, to the night dew, and to wet and moisture, ought carefully to be avoided; and if at any time these exposures are inevitable, the system should be fortified against their effects; but the mode of fortifying the system requires consideration. This should not be attempted, unless better means are not within reach, by wines or spirits: these generally leave the system, as soon as their stimulating effects have passed off, more exposed than before to the invasion of disease. Permanent tonics, however, and those more especially which determine to the surface of the body, at the same time that they improve the tone of the digestive viscera, and promote the regular functions of the bowels and biliary organs, may be resorted to on such occasions. For this purpose, infusion or decoction of bark, or of calumba, may be taken with the spiritus Mindereri, or any warm stomachic; or the powdered bark may be exhibited, combined with the spicy aromatics. The same medicinal means may be also attended to, whenever the disease prevails at the place where the individual resides, and should be put in practice when he retires to sleep, and as soon as he rises in the morning, before he leaves his apartment. He should avoid, also, sleeping in low and ill-ventilated apartments; and be equally distrustful of sleeping near, or even of passing through, in the night time, marshy or swampy districts. If, however, these latter precautions cannot be taken, the medicinal means already suggested should be adopted.

"The bowels should be attended to, and their functions regulated; but in no case should this be attempted by debilitating purgatives, or by salts. The warm stomachic laxatives, and these combined with tonics, may be adopted with advantage, as occasion may require. The surface of the body should be kept in a warm perspirable state; but excessive perspirations must be avoided.

"The diet should be regular, moderate, and easy of digestion. Whilst low living ought to be shunned, its opposite should never be indulged in. The stomach ought to have no more to do than what it can perfectly accomplish, without fatigue to itself, and to the promotion of its own energies. It must never be roused to a state of false energy, by means of palatable excitants, or weakened by distending it with too copious draughts of weak diluents.

"The state of the mind ought to be regulated in such a manner as not to be excited much above, or lowered beneath, its usual tenor. The imagination should not be allowed, for a moment, to dwell upon the painful considerations which the disease is calculated to bring before the mind; and least of all ought the dread of it to be encouraged. There is a moral courage, which is possessed by individuals who are even the weakest, perhaps, as respects physical powers, and which in them resists more efficiently the causes of intertropical diseases, than the bodily powers of the strongest, who are not similarly endowed with this species of mental energy.

"Those who dread not the attack of disease, more especially of epidemic disease, and who yet possess sufficient prudence to avoid unnecessary exposure to their predisposing and exciting causes, may generally be considered as subjected to comparatively little risk from them. This, I am persuaded, is particularly the case as respects epidemic cholera, and I wish to impress it upon the minds of those whom the observation concerns." (P. 8.)

Upon the subject of the preservative effects of *fumigations*, Dr. Hawkins states that the observations of those who have witnessed the disease in Russia are not very promising. Such agents may extinguish a bad smell, by presenting one of a stronger or more agreeable kind, or they may even, to a certain extent, purify the atmosphere; but we have, as yet, no reason to believe that they can intercept the passage of contagion from one individual to another. A free ventilation will probably be found everywhere more useful, both to the public at large, to the sick, and their attendants. Dr. Albers, who was sent to Russia, at the head of a medical commission, by the Prussian government, states in his report, that fumigations of vinegar and chlorine had an ample trial, but appeared to be useless: chlorine, indeed, was worse than useless, as, when employed in excess, it seemed to do harm. He remarks, that at the very time when the hospital was filled with the clouds of chlorine, the largest proportion of the attendants was attacked.

Dr. Hawkins gives an interesting sketch of the early history of the spasmodic cholera of India, and he also describes the symptoms by which it was characterised, and the method of cure that was adopted, in the more fatal form and more extensive range which it began to assume in 1817. No marked peculiarity of weather was observed previously to its appearance; and it does not appear to have been at all affected in its severity or progress by the circumstances of season, temperature, or moisture: "it was observed to have prevailed with equal violence when the thermometer was at 40° or 50°, as when it stood at 90° or 100°; during the prevalence of incessant rains for months, and when the face of the earth was scorched by long-continued heat and drought. The disease was very uniform in its principal features, and attacked, almost indiscriminately, people of all nations and constitutions, both Europeans and natives. These circumstances, as well as some striking features of the disease, induced the Board of Bombay to consider the name of cholera morbus as inapplicable, which we believe to be a general opinion."

As many have doubted the identity of the Russian and Indian cholera, it may be proper to state that all the Russian official reports unite in shewing that the disease is one and the same. Dr. Ashburner and Mr. Searle, both of whom have seen and felt the effects of the disease, concur in this opinion of the Russian authorities.

"We perceive that nearly the same remedies have been everywhere adopted. In Persia, M. Gamba informs us that bleeding, calomel, laudanum, and preparations of ether, were the medicines employed. Everywhere also the disease has committed its principal ravages amongst the poorer inhabitants, amongst those who are subjected to inclemencies of weather, who are compelled to use severe labour for their daily support; who obtain a scanty and often improper nourishment; who are lodged in damp and ill-ventilated abodes, and whose clothing is insufficient and seldom changed. The camp followers of our Indian army seem to have been most fatally affected by the epidemic; after them, in the gradation of suffering, follow the native troops; then come the English common soldiery, then the English officers, and, last of all, are found the civilians. Women appear less subject to it than men, and children less subject than adults. In Europe we perceive a similar order of exposure and liability. Everywhere in Europe the lower orders have been the chief sufferers: the number of remarkable persons and of nobility who have fallen victims has been extremely small; their names, indeed, might be easily enumerated. When such instances have occurred among the affluent classes, they might probably be explained by particular mental anxiety, or a state of predisposing bad health. The soldiers of the conflicting armies in Poland have been amongst the chief sufferers: we may easily imagine the privations to which they have been exposed. At Warsaw the Committee of Health announce, that very few persons of easy condition have been ill, and that the disease has expended its chief ravages on the poorer inhabitants of the low and thickly-peopled quarter of the city, which lies near the Vistula. At Riga, the sailors appear to have been most liable to the disorder.

"One of the first circumstances which strikes us in the history of this disorder, is the name it has acquired, the term cholera seeming to imply that it consists of a redundancy or depravity of the bile; whereas it appears that the secretion and excretion of the bile are entirely suspended, and that the matter evacuated by vomiting and purging is quite of a different character. This is an inaccuracy, however, into which the ancients, as well as the moderns, have fallen, and is best elucidated by Alexander Trallian. This ancient author describes three species of cholera. In the most intense, there is no evacuation of bile; and he thinks the name might be more properly derived from χολαδες, an old Greek word used by Homer to signify the bowels, than from χολη, bile. In the species next in degree, however, he says there is a great discharge of bile, and being attended with excruciating spasms like the former, obtains the same name. The third species is a simple bilious diarrhœa, without the spasms. In the disease as it occurs in ordinary practice in this country, most commonly in the month of August, one of the most prominent symptoms is certainly the discharge of a large quantity of bile, and it seems to be the middle species of Trallian." (P. 123.)

The common *bilious cholera* is a disease distinct from the spasmodic cholera. In India, as well as in Europe, the former is a disease well known, and not usually fatal.

"In the first, as the name indicates, the discharges are more or less coloured with bile, and the general commotion of the alimentary canal seems to arise from a superabundance of bile thrown upon it. In the spasmodic cholera, on the contrary, the discharges are generally whitish, and no hope is held out of recovery until they acquire a bilious tinge." (P. 127.)

The common bilious cholera is sometimes epidemic.

Upon the subject of the contagious nature of the disease, much contrariety of opinion has, and still continues to exist. Dr. Hawkins very pertinently asks, whether we can have a better proof of the contagious nature of the disease, than that *insulation*, or separation from the sick, is almost universally found to preserve from the evil?

"Let us hear the history of Mr. de Lesseps, the consul of France at Aleppo, an individual who probably has never interfered in medical discussions. When the cholera approached that city in 1822, this gentleman retired, in company with all who wished to be of his party, to a garden at some distance from the city. His asylum was enclosed with walls, and was surrounded by a large fossé: there were only two doors, one for entrance, the other for going out. As long as the malady lasted, he admitted nothing from out of doors without submitting it to the precautions observed in lazarettoes. His colony comprised two hundred persons, and consisted not only of Franks more or less acclimatised, but also of several natives. Not a single individual contracted the disease; while, at the very same time, within the city, four thousand beings perished in the space of eighteen days.*" (P. 151.)

After adverting to different opinions upon this very important point, Dr. Hawkins very truly remarks, that the middle course of reasoning is, on this subject, probably the true, as well as the safe one. There is no necessity for embracing either extreme, for becoming entirely a contagionist or an anti-contagionist. We may easily conceive that a disease may originally be produced by local or other causes, which may not be endued with a self-communicating power at its birth, but which may nevertheless acquire the contagious element as it proceeds and gathers strength through the new circumstances which occur in its passage.

The Appendix to Dr. Hawkins' work contains many interesting documents respecting the disease as it has existed in Russia and India, together with various communications

* P. F. Kerandran, Mémoire sur le Choléra Morbus de l'Inde. Paris, 1831.

upon the subject which have been published by English and foreign physicians.

Mr. AUSTIN's pamphlet is addressed more to the general than the professional reader: his objects are to place before the public a short account of the history, nature, and symptoms of the disease, divested as much as possible of technicalities, and to point out the most reasonable mode of prevention, or rather precautions, necessary to be observed; and in case of attack, the mode of treatment experience has proved to be the most judicious.

